# Oral post-exercise garlic extract supplementation enhances glycogen replenishment but does not up-regulate mitochondria biogenesis mRNA expression in human-exercised skeletal muscle

**DOI:** 10.1080/15502783.2024.2336095

**Published:** 2024-04-04

**Authors:** I-Shiung Cheng, Jung-Piao Tsao, Jeffrey R. Bernard, Tsen-Wei Tsai, Chia-Chen Chang, Su-Fen Liao

**Affiliations:** a National Taichung University of Education, Department of Physical Education, Taichung City, Taiwan; b Department of Sports Medicine, China Medical University, Taichung City, Taiwan; c Department of Kinesiology and Public Health Promotion,California State University, Stanislaus, Turlock, USA; d Department of Nursing, Taichung, China Medical University Hospital, Taiwan; e College of HuilanNational Dong Hwa University, Physical Education Center, Hualien, Taiwan; f Department of Physical Medicine and Rehabilitation, Changhua Christian Hospital, Changhua, Taiwan; g Department of Post-Baccalaureate Medicine, College of Medicine, National Chung Hsing University, Taichung, Taiwan

**Keywords:** Ergogenic property, phytochemicals, exercise, muscle adaptation

## Abstract

**Purpose:**

Garlic extract (GA) is purported to enhance antioxidant and anti-inflammatory activity and glucose regulation in humans. The present study investigated the effects of post-exercise GA supplementation on GLUT4 expression, glycogen replenishment, and the transcript factors involved with mitochondrial biosynthesis in exercised human skeletal muscle.

**Methods:**

The single-blinded crossover counterbalanced study was completed by 12 participants. Participants were randomly divided into either GA (2000 mg of GA) or placebo trials immediately after completing a single bout of cycling exercise at 75% Maximal oxygen uptake (VO_2max_) for 60 minutes. Participants consumed either GA (2000 mg) or placebo capsules with a high glycemic index carbohydrate meal (2 g carb/body weight) immediately after exercise. Muscle samples were collected at 0-h and 3-h post-exercise. Muscle samples were used to measure glycogen levels, GLUT4 protein expression, as well as transcription factors for glucose uptake, and mitochondria biogenesis. Plasma glucose, insulin, glycerol, non-esterified fatty acid (NEFA) concentrations, and respiratory exchange ratio (RER) were also analyzed during the post-exercise recovery periods.

**Results:**

Skeletal muscle glycogen replenishment was significantly elevated during the 3-h recovery period for GA concurrent with no difference in GLUT4 protein expression between the garlic and placebo trials. PGC1-α gene expression was up-regulated for both GA and placebo after exercise (*p* < 0.05). Transcript factors corresponding to muscle mitochondrial biosynthesis were significantly enhanced under acute garlic supplementation as demonstrated by TFAM and FIS1. However, the gene expression of SIRT1, ERRα, NFR1, NFR2, MFN1, MFN2, OPA1, Beclin-1, DRP1 were not enhanced, nor were there any improvements in GLUT4 expression, following post-exercise garlic supplementation.

**Conclusion:**

Acute post-exercise garlic supplementation may improve the replenishment of muscle glycogen, but this appears to be unrelated to the gene expression for glucose uptake and mitochondrial biosynthesis in exercised human skeletal muscle.

## 1. Introduction

Garlic supplementation has been reported to have positive effects on exercise performance [1]. Garlic extracts (GA) in particular are known to contain a multitude of products that have a physiological impact on humans [2]. For example, GA contains phytochemicals such as flavonoids and sulfur compounds. GA also contains biologically active compounds including 1-propenyl allyl thiosulfonate, allyl methyl thiosulfonate, (E,Z)-4,5,9-trithiadodeca-l,6,11-triene 9-oxide (ajoene), y-L-glutamyl-S-alkyl-L-cysteine, etc [3]. Due to these products, GA has been shown to improve glucose regulation [4], antioxidant capacity, blood pressure, cholesterol as well and athletic performance [5,6]. A meta-analysis by Hou et al. found that garlic reduces fasting blood glucose levels based on 7 published human studies, including 5 studies involving patients with type II diabetes and 2 studies involving healthy subjects at rest [7]. Despite this evidence, the effects of garlic supplementation on blood glucose regulation on the general population and athletes remain to be fully elucidated. Yang et al. found that the expression of glucose transporter protein (GLUT4) was increased in muscle tissue in animal models supplemented with garlic, with S-allylcysteine sulfoxide (SACS) in GA potentially playing a significant role in this effect [8]. Furthermore, animal studies have demonstrated that a single dose of SACS at a dosage of 200 mg/kg body weight improves oral glucose tolerance, indicating the potential involvement of GA in regulating blood glucose levels [9]. However, there are few studies investigating oral GA supplementation to improve glucose metabolism in humans.

GLUT4, located in skeletal muscle, is responsible for regulating glucose entry into muscle for energy utilization. Moreover, studies in humans have demonstrated that the expression of GLUT-4 mRNA was up-regulated and a significant increase in protein expression of GLUT-4 was observed post-exercise. [10]. A human study conducted by Richter et al. demonstrated that high expression of GLUT4 significantly increased whole-body glucose utilization and contributed to the synthesis of glycogen in skeletal muscle and liver [11]. Surprisingly, previous human studies examining the effects of ergogenic aids on exercise have shown inconsistent findings regarding the beneficial influence of GLUT4 expression and glycogen synthesis in muscle and liver during recovery [12–14]. To date, no studies have examined the effect of garlic extract on GLUT4 expression in exercised human skeletal muscle.

PPARγ coactivator-1α (PGC-1α), nuclear respiratory factors (NRFs), and mitochondrial transcription factor A (TFAM) are important regulators of mitochondrial biosynthesis in the body [15]. Moreover, PGC-1α serves as a physiological signal that up-regulates nuclear respiratory factors (NRFs), which are nuclear transcription regulatory factors, and it also activates mitochondrial transcription factor A (TFAM). TFAM is a mitochondrial biosynthesis gene that is located in the mitochondrial membrane, and regulates factors involved in the replication and transcription of mitochondrial DNA (mtDNA) [16]. In addition, the biosynthesis of cellular mitochondria is regulated by various physiological factors such as the saturation or deficiency of body energy, the level of oxidative stress caused by reactive oxygen species (ROS), and the body’s capacity to store exogenous food energy [17]. Thus, the physiological stress induced by exercise can affect the regulation of cellular PGC-1α in mitochondrial fusion and fission, which includes the gene expression of Mfn1, Mfn2, Opa1, Drp1, and FIS1 [18]. Therefore, we hypothesized that GA will dampen the exercise-induced purported antioxidant capacity. The expression of PGC-1α significantly increases in response to intense exercise in humans, as demonstrated by studies conducted by Pilegaard et al. [19] and Huang et al. [20]. However, the upstream targets of mitochondrial biosynthesis regulatory factors, such as NRF1, NRF2, and TFAM genes, were not affected by the exercise [20]. Oral ergogenic supplements consumed post-exercise are popular among elite athletes for recovery purposes [21]. Despite this, whether the exercise-induced benefit on mitochondrial biogenesis of muscle cells can be dampened by post-exercise oral antioxidant phytochemical supplementation remains unclear. Collectively, we hypothesized that acute post-exercise GA supplementation would enhance muscle GLUT4 expression and glycogen replenishment. It’s also hypothesized that supplementation would influence mitochondrial biogenesis-related fusion/fission transcript factors throughout the recovery period. Therefore, we investigated the ergogenic properties of GA on muscle GLUT4 and glycogen levels, as well as transcript factors involved with mitochondrial synthesis in young active adults.

## 2. Methods

### 2.1. Participants

Twelve physically active male university students were recruited and voluntarily participated in this study. Prior to the formal experiment, a pre-trial was conducted to measure their maximal oxygen consumption. If any discomfort was experienced during the pre-trial, it would be stopped immediately, and the participant would not continue to the formal experiment. The experimental protocol was conducted with the approval of the Institutional Review Board at the University of Taipei, in Taiwan (IRB-2019-074). After explaining the experimental protocol of the study, all participants signed the consent form to participate in this study. During the study, participants were allowed to voluntarily withdraw themselves at any time without reason.

### 2.2. Experimental protocol

The present investigation utilized a single-blind and crossover counterbalanced study design. Twelve healthy college-aged male students were recruited and participated in this study. The participants had an average age of 21.4 ± 3.3 years, height of 175.0 ± 8.8 cm, weight of 75.6 ± 6.4 kg, and VO_2max_ of 46.9 ± 7.3 ml/kg/min. Each participant underwent two trials with a period of seven days between them, allowing for a 7-day washout period. Seven days prior to each trial, the participant’s VO2max was determined, and their maximum wattage required to maintain 60 revolutions per minute (60 rpm) during a single bout of cycling ergometer (Monark 894 E, Varberg, Sweden) at 75% VO2max was calculated. Three days prior to the experiment day, participants were required to adhere to dietary restrictions and abstain from coffee and smoking. Participants were instructed to fast for 12-hour prior their bout of exercise. On the day of the experiment, they arrived at the laboratory at 7:00 am and rested for 5 minutes before performing a 60-min cycling exercise at 75% VO_2max_ while maintaining a cadence of 60 rpm [14]. They were given the opportunity to drink water ad libitum and take a shower within 10 minutes after completing their ride. Immediately after the 60-min cycling exercise, subjects received a high glycemic index carbohydrate meal, equivalent to 2 g/kg of body weight, and ingested a capsule containing either 2000 mg of GA or placebo. The food composition in the meal includes Corn Flakes (Kellogg’s Ltd, Manchester, UK), skimmed milk, white bread, strawberry jam and glucose water with an overall glycemic index of 76.6. At least 7 days after completing the first trial, each participant performed their second trial (i.e. just changing the oral ergogenic aid) while maintaining the single-blind and crossover counterbalanced design. Blood and gas samples were collected before, during, and 3-h after exercise. Muscle samples were collected immediately after (0 h) and 3-h post-exercise for periodic acid-Schiff (PAS) stain and immunohistochemistry (IHC) analysis, measuring muscle glycogen resynthesis and protein expression of GLUT4, ATP Citrate Lyase. The expression of genes involved in muscle mitochondrial biogenesis, including PGC-1α, SIRT1, ERRα, NFR1, NFR2, TFAM, MFN1, MFN2, OPA1, Beclin-1, DRP1, FIS1, and genes involved in muscle glucose utilization, including TBC1D1, TBC1D4, GLUT4, and HKII, were quantified by real-time PCR. Muscle samples were obtained from the vastus lateralis muscle, with a distance of at least 2 cm between sampling sites. Blood samples were collected before and every 30 min during the post-exercise period of 0-h to 3-h. Gas samples were collected for every 10 min at 0-, 1-, 2-, and 3-h post exercise using the MetaMax system (Leipzig, Germany). Blood samples were also analyzed for concentrations of glucose, insulin, free fatty acid, and glycerol, while gas samples were analyzed for fatty acid oxidation rates.

### 2.3. Biopsy muscle sample collection

The muscle biopsy was performed under one to two mL of anesthetic (2% lignocaine) injection for local anesthesia. Subsequently, the no. 14 muscle puncture needle (Temno, McGaw Park, IL, USA) was used to puncture the lateral femoral muscle of the participants to obtain muscle samples (approximately 20–50 mg) [22]. Any residual blood, fat, and connective tissue were removed from the muscle. Muscle samples were then blotted dried, flash frozen in liquid nitrogen, and stored at −80 degrees C until analyzed.

### 2.4. Muscle samples and Immunohistochemistry (IHC)

Samples from the lateral side of the thigh muscle were collected via a muscle biopsy. Samples were immediately soaked in a small glass test tube containing 20 ml of formalin (10%). The tissue was then dehydrated using a tissue processor and embedded in paraffin blocks. After 24–48-h of fixation in 10% formalin, the muscle tissue was sectioned and mounted on adhesive slides suitable for IHC staining. The staining protocol was performed on the Leica Bond Max automated immunohistochemical staining machine. For antibody staining, the antibody was diluted, and antigen retrieval was performed using Bond EDTA buffer (pH 9) at 100°C for 20 minutes. The primary antibodies used were as follows: GLUT4 (bs-0384, BIOSS, Beijing, China) at a 1:200 dilution, ATP citrate lyase (ab40793, Abcam, Cambridge, MA, USA) at a 1:500 dilution, Citrate synthetase (ab129095, Abcam, Cambridge, MA, USA) at a 1:500 dilution.

### 2.5. Periodic Acid-Schiff staining (PAS)

Skeletal muscle samples embedded in paraffin were sectioned for periodic acid-Schiff staining (PAS). PAS was used to detect muscle glycogen levels. The paraffin section was cut 3 um under the processes including 1) deparaffinize and hydrate to water; 2) oxidize in 0.5% periodic acid solution for 5 minutes; 3) wash in tap water for 5 minutes; 4) rinse in distilled water twice; 5) place in Schiff reagent for 15 minutes (Sections become light pink color during this step); 6) wash in tap water for 10 minutes (Immediately sections turn dark pink color); 7) counterstain in light green for 10 seconds; 8) rinse in distilled water for 1 second; 9) dehydrate and coverslip using a synthetic mounting medium. The sections were observed under a microscope (BX53, Olympus, Tokyo, Japan) at 200× magnification, and images captured using a microscope digital imaging system camera (DP21, Olympus, Tokyo, Japan). The histological sections were processed and interpreted by the pathology laboratory at National Chung Hsing University.

### 2.6. Gene expression analysis

Real-time RT-PCR was used to analyze gene expression in muscle samples. RNA was extracted from the tissue using the TRIzol kit (Life Technologies, Rockville, MD, USA). Using a spectrophotometer, absorbance was measured at 260 nm, and the concentration of total RNA per µL of solution was calculated. A 200 ng sample of RNA was used to prepare cDNA via a reverse transcription (RT) reaction using a high-capacity cDNA reverse transcription kit (Applied Biosystems, Foster City, CA, USA). The RT reaction mixture included an RT buffer, dNTP mix, RT random primers, MultiScribe^TM^ reverse transcriptase, and nuclease-free H_2_O. The RT reaction was performed at 25℃ for 10 minutes, 37℃ for 120 minutes, and 85℃ for 5 seconds, followed by cooling to 4℃. The cDNA was then subjected to real-time RT-PCR/SYBR Green using the standard method provided by Applied Biosystems (Foster City, CA, USA). The Power SYBR Green PCR Master Mix (Applied Biosystems, Foster City, CA, USA) was used, with each sample run in duplicate on the StepOne^TM^ real-time RT-PCR System. SYBR Green was set to be reporter dye as the detector for each sample. Subsequently, the RT-PCR reaction was carried out for 40 cycles at 95℃ for 10 minutes, 95℃ for 15 seconds, and 60℃ for 1 minute [23]. The fluorescence signal produced by SYBR Green binding to the amplified product was analyzed using the system software. Primer pairs for measuring were found in Table 1.

**Table 1.** Primer pairs for measuring in the quantitative RT-PCR assay.

Gene name	(Forward)	GenBank NO.
GLUT4	Forward 5’-CACAGTCTTCACCTTGGTCTCG-3’ Reverse 5’-GTAGCTCATGGCTGGAACTCG-3’	GI: 6517
HKII	Forward 5’-TTGTCCGTAACATTCTCATCGATT-3’ Reverse 5’-TGTCTTGAGCCGCTCTGAGAT-3’	GI: 3099
TBC1B1	Forward 5’-GTGTGGGAAAAGATGCTTAGCA-3’ Reverse 5’-GTGATGACGTGGCACACCTT-3’	GI: 23216
TBC1B4	Forward 5’-AGCTCCAGTGAACAGTGCAGTG-3’ Reverse 5’-CACTTAGGGACTCATTGCTGC-3’	GI: 9882
PGC-1α	Forward 5’-CGAGGAATATCAGCACGAGAGG-3’ Reverse 5’-CATAAATCACACGGCGCTCTTC-3’	GI: 10891
SIRT1	Forward 5’-TACGACGAAGACGACGACGA-3’ Reverse 5’-CGCCGCCGCCGCCTCTTCC-3’	GI: 23411
ERRα	Forward 5’-TGCCAATTCAGACTCTGTGC-3’ Reverse 5’-CCAGCTTCACCCCATAGAAA-3’	GI: 2101
NRF1	Forward 5’-CTACTCGTGTGGGACAGCAA-3’ Reverse 5’-AGCAGACTCCAGGTCTTCCA-3’	GI: 4899
NRF2	Forward 5’-AAGTGACAAGATGGGCTGCT-3’ Forward 5’-TGGACCACTGTATGGGATCA-3’	GI: 4780
TFAM	Forward 5’-CGCTCCCCCTTCAGTTTTGT-3’ Reverse 5’-CACTCCGCCCTATAAGCATC-3’	GI: 7019
Beclin-1	Forward 5’-AAGATTGAAGACACTGGAGGCA-3’ Reverse 5’-GAGGACACCCAAGCAAGACC-3’	GI: 8678
DRP1	Forward 5’-AGGTTGCCCGTGACAAATGA-3’ Reverse 5’-ATCAGCAAAGTCGGGGTGTT-3’	GI: 100559
FIS1	Forward 5’-GTCCAAGAGCACGCAGTTTG-3’ Reverse 5’-ATGCCTTTACGGATGTCATCATT-3’	GI: 51024
MFN1	Forward 5’-ATGACCTGGTGTTAGTAGACAGT-3’ Reverse 5’-AGACATCAGCATCTAGGCAAAAC-3’	GI: 55669
MFN2	Forward 5’-CACATGGAGCGTTGTACCAG-3’ Reverse 5’-TTGAGCACCTCCTTAGCAGAC-3’	GI: 9927
OPA1	Forward 5’-AGCCTCGCAATTTTTGG-3’ Reverse 5’-AGCCGATCCTAGTATGAGATAGC-3’	GI: 4976

### 2.7. Blood sampling and analysis

Blood samples (5 ml) were collected using a 20 G Jelco needle (Tampa, FL, USA) at designated time points (as outlined above) before, during, and after exercise. Blood samples were collected in vacuum tubes or tubes containing fluoride heparin and then centrifuged at 1000 × g for 10 minutes. The resulting supernatant was stored at −80℃ for later analysis. The concentration of blood glucose was analyzed using an automated glucose oxidase method (YSI Life Sciences, Yellow Springs, OH, USA), while free fatty acids (Wako Chemicals GmbH, Wako, Neuss, Germany) and insulin (Baylor Diagnostics, Tarrytown, NY, USA) concentrations were determined using immunoassay methods.

### 2.8. Oxidation rate of carbohydrate and fat

Gas analysis was performed every 10 min post exercise using a Metamax 3B system (Cortex Biophysik, Nonnestrasse, Leipzig, Germany) to measure the concentrations for both oxygen consumption (VO2) and validation of carbon dioxide production (VCO2) [24]. The respiratory quotient (RQ) was determined, and oxidation rates of carbohydrate and fat were calculated using the following equations [25,26]:

Carbohydrate oxidation rate (g/min) = 4.585 × VCO_2_ − 3.226 × VO_2_

Fat oxidation rate (g/min) = 1.695 × VO_2_ − 1.701 × VCO_2_

### 2.9. Statistical analysis

All experimental data were reported as mean ± standard deviation (mean ± SD). SPSS software was used for statistical analysis. 12 subjects recruited were used to achieve 80% power at the significant level of 5%. A paired t-test was used to compare resynthesized glycogen, expression of genes involved in glucose uptake, and relevant gene expression of mitochondrial biosynthesis between the two trials. A repeated-measures two-way ANOVA (with factors for trial and time) was used to analyze changes in blood and gas data over time within groups. When there was a significant interaction between the experimental procedure and time point, a simple main effects analysis was conducted. Post-hoc analysis was performed using Fisher’s least significant difference test. The level of statistical significance was set at alpha = 0.05.

## 3. Results

### 3.1. Post-exercise garlic extract supplementation improved blood glucose influx and the replenishment of muscle glycogen which was independent of insulin action

**Figure 1.**
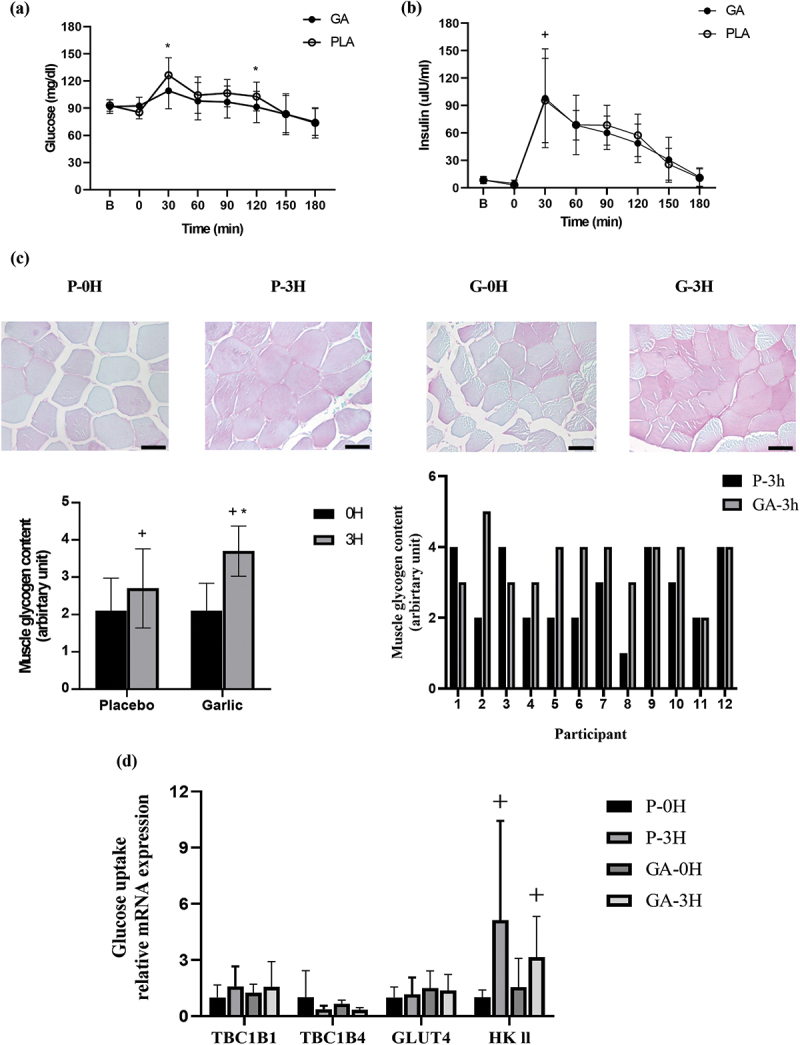
Blood glucose (a), insulin concentrations (b), glycogen content (c), and glucose uptake-related gene expression (d) after a single bout of exercise in GA (-•-) and PLA (-○-) trials. GA: garlic; PLA: placebo; *p*-0 H: immediately after exercise in the placebo trial; *p*-3 H: 3 h after exercise in the placebo trial; G-0 H: immediately after exercise in garlic trial; G-3 H: 3 h after exercise in garlic trial. Values are expressed as mean ± SD, N = 12. *significant difference against placebo at the same time (*p* < 0.05). ^+^significant difference against 0 h (immediately after exercise; *p* < 0.05).

Figure 1a shows that plasma glucose concentrations were significantly lower in the garlic trial compared to that in placebo at both the 30-minute and 120-minute time points during the exercise recovery period (*p* < 0.05, 1A). Despite differences in blood glucose, there was no difference in insulin response between post-exercise garlic and placebo trials. Of interest, muscle glycogen levels, as seen by PAS staining, were significantly enhanced after garlic extract supplementation compared to that in the placebo trial (Figure 1c *p* < 0.05). The data for individual muscle glycogen levels after post-exercise placebo/garlic supplementation was referenced in Table 2. The data on individual muscle glycogen levels after exercise showed that 7 subjects increased at 3 hours compared to 0 hours, 2 subjects decreased, and 3 subjects remained at the same level. GLUT4 mRNA (Figure 1d) and protein expression (Figure 4c), which were estimated by RT-PCR and immunohistochemistry (IHC) staining, were not improved after post-exercise garlic supplementation. However, HKII gene expression was higher at 3 h compared to 0 h during the exercise recovery period in both garlic and placebo trials. It was noted that post-exercise garlic supplement was unable to influence whole-body insulin action during the 3-h exercise recovery period, However, the human exercised muscle glycogen replenishment was enhanced after acute post-exercise garlic extract supplementation.

**Table 2.** Individual data of muscle glycogen during post-exercise recovery in placebo and garlic trials (arbitrary unit).

Group	Placebo			Garlic		
Time (hour)	0 h		3 h	0 h		3 h
Subject 1	2.0		4.0	2.0		3.0
Subject 2	1.0		2.0	3.0		5.0
Subject 3	3.0		4.0	1.0		3.0
Subject 4	1.0		2.0	3.0		3.0
Subject 5	2.0		2.0	2.0		4.0
Subject 6	3.0		2.0	2.0		4.0
Subject 7	3.0		3.0	3.0		4.0
Subject 8	1.0		1.0	1.0		3.0
Subject 9	2.0		4.0	2.0		4.0
Subject 10	3.0		3.0	2.0		4.0
Subject 11	1.0		2.0	1.0		2.0
Subject 12	2.0		4.0	2.0		4.0

### 3.2. Post-exercise garlic extract supplementation slightly increased carbohydrate oxidation throughout the recovery period

**Figure 2.**
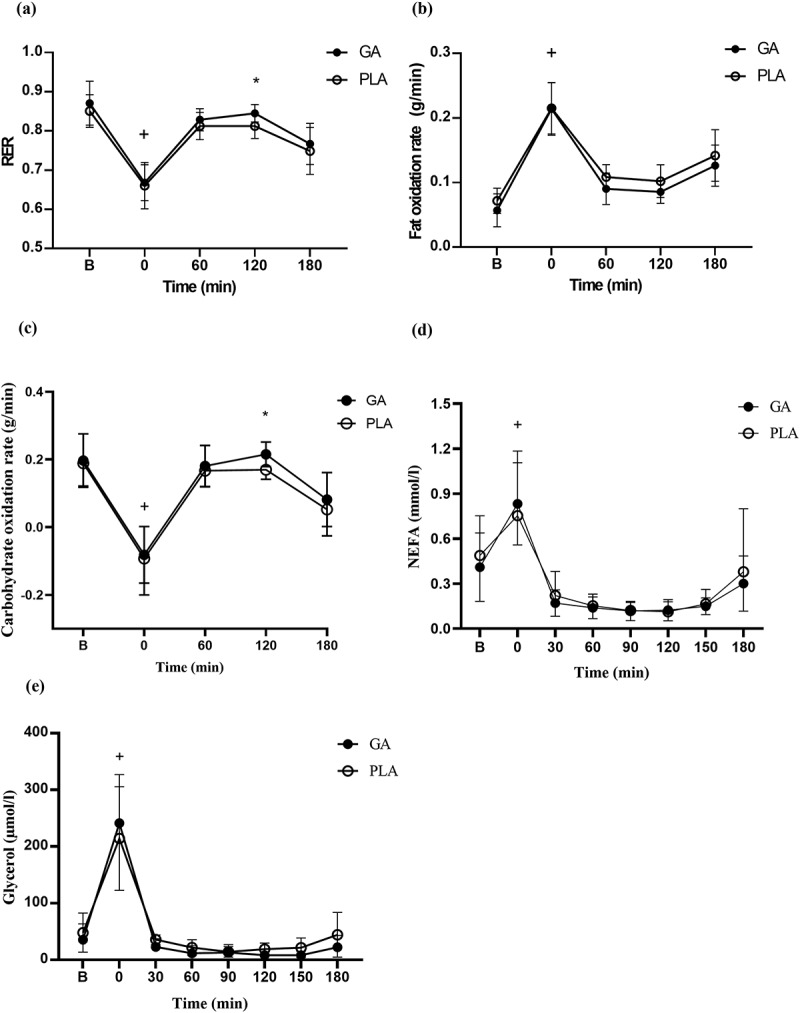
Respiratory exchange ratio (RER) (a), fat oxidation rate (b), carbohydrate oxidation rate (c), non-esterified fatty acid (NEFA) (d), and glycerol (e) concentrations after a single bout of exercise in GA (-•-) and PLA (-○-) trials. GA: garlic; PLA: placebo. Values are expressed as mean ± SD, *N* = 12. *significant difference against placebo at the same time (*p* < 0.05). ^+^ significant difference against B h (immediately after exercise; *p* < 0.05).

All gaseous samples from each 10-minute period during exercise recovery were averaged, including RER, VO2, VCO2, carbohydrate oxidation rate, and fat oxidation rate during post-exercise recovery in both the placebo and garlic trials, as shown in Table 3. Figure 2 shows changes in respiratory exchange ratio (RER) (Figure 2a), fat oxidation rate (Figure 2b), carbohydrate oxidation rate (Figure 2c), plasma non-esterified fatty acids (NEFA) (Figure 2d), and glycerol (Figure 2e) during the post-exercise recovery period in both garlic and placebo trials. In regard to RER, the general trend suggested that garlic extract significantly increases RER at 120 minutes (Figure 2a), and showed higher carbohydrate oxidation rates (Figure 2c). Based on gas exchange data, the calculated fat oxidation rate showed no significant differences between the two trials during the 3-h post-exercise period (Figure 2b). As for lipolysis, the levels of plasma NEFA in the garlic trial were similar to that of the placebo trial (Figure 2d). In addition, there were no significant responses for the expressions of ATP Citrate Lyase (Figure 4a), and Citrate synthetase (Figure 4b) observed for the garlic trial.

**Table 3.** The values of RER, VO_2_, VCO_2_, CHO oxidation rate, and fat oxidation rate during post-exercise recovery in placebo and garlic trials.

Group		Placebo						Garlic					
Time (min)		60 min		120 min		180 min		60 min		120 min		180 min	
		Mean	SD	Mean	SD	Mean	SD	Mean	SD	Mean	SD	Mean	SD
RER VO_2_ (ml/kg/min) VCO_2_ (ml/kg/min)		0.81 0.35 0.28	0.03 0.05 0.04	0.81 0.32 0.26	0.03 0.07 0.06	0.74 0.32 0.24	0.05 0.06 0.39	0.83 0.32 0.26	0.03 0.08 0.07	0.85* 0.33 0.28*	0.02 0.06 0.05	0.77 0.32 0.25	0.05 0.05 0.05
CHO oxidation rate (g/min)		0.17	0.05	0.16	0.05	0.05	0.08	0.18	0.06	0.21*	0.04	0.09	0.08
FAT oxidation rate (g/min)		0.11	0.02	0.10	0.02	0.14	0.04	0.09	0.02	0.09	0.02	0.13	0.03

*Significant difference against at the same time. (*p < 0.05).

### 3.3. PGC-1α was activated by exercise for both the garlic and placebo trials, but post-exercise acute garlic supplementation did not upregulate the mRNA expression for mitochondria biogenesis or energy metabolism

**Figure 3.**
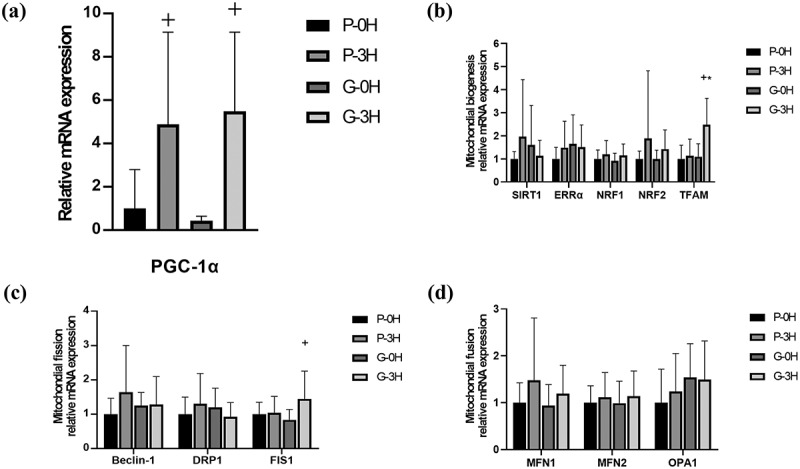
PGC-1α (a), mitochondrial biogenesis-related gene expression (b), mitochondrial fission-related gene expression (c), and mitochondrial fusion-related gene expression (d) in vastus lateralis of human skeletal muscle after a single bout of exercise in GA and placebo trials. Values are expressed as mean ± SD, *N* = 12. *significant difference against placebo at the same time (*p* < 0.05). ^+^significant difference against 0 h (immediately after exercise; *p* < 0.05).

**Figure 4.**
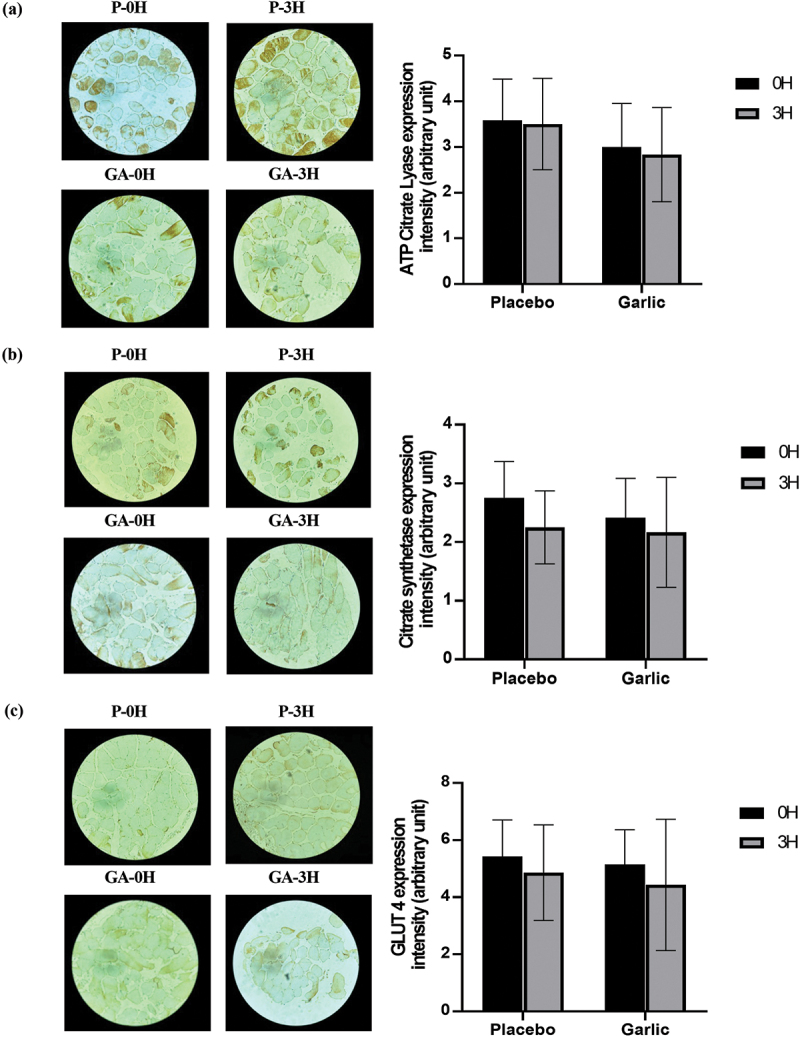
Representative pictures of various immunostaining patterns of ATP citrate lyase (a), citrate synthetase (b), and GLUT4 (c) expression intensity (arbitrary unit) in vastus lateralis of human skeletal muscle after a single bout of exercise in GA and placebo trials. The stained tissues were observed under a bright-field microscope with 200X (muscle tissue) magnification. Values are expressed as mean ± SD, *N* = 12.

Figure 3a shows the influence of garlic extract on PGC-1α expression and muscle cell mitochondrial biogenesis-related gene expression including for SIRT1, ERRα, NFR1, NFR2, TFAM (Figure 3b), and Beclin-1, DRP1, FIS1, MFN1, MFN2, OPA1 (Figure 3(c,d)). In both trials, PGC-1α gene expression was significantly higher at 3 h post-exercise compared to that immediately after exercise (0 h). As displayed in Figure 3(b,c), there was a significant difference in TFAM and FIS1 among the two trials. Nevertheless, no significant differences were found in the mitochondrial biogenesis-related expressions of SIRT1, ERRα, NFR1, NFR2, Beclin-1, DRP1, MFN1, MFN2, OPA1 during the exercise recovery period between garlic and placebo trials (Figure 3(b–d), *p* > 0.05). Additionally, there was no significant change in protein expression of GLUT4 (Figure 4c) during the recovery period after acute garlic supplementation.

## 4. Discussion

The aim of the present investigation was to evaluate the effects of a high-dose (2000 mg) oral GA supplementation consumed immediately post-exercise on skeletal muscle energy replenishment. Experimental samples including muscle tissue, blood, and gas were collected to assess exercise physiology-related changes. The study’s findings demonstrated that: 1) oral GA supplementation improved muscle glycogen replenishment post-exercise, in the absence of improvements in whole-body insulin sensitivity; 2) slightly increased the higher exercise-induced carbohydrate oxidation with GA supplementation; and 3) PGC-1α gene expression was significantly activated by exercise, but not affected by oral garlic supplementation as it relates to upregulating genes associated with muscle cell mitochondrial biosynthesis. To the best of our knowledge, this is the first human study to demonstrate that high-dose GA supplementation during the recovery period can improve the ability of muscle glycogen replenishment. However, it does not appear to upregulate the expression of transcription factors involved in skeletal muscle mitochondrial biosynthesis.

Interestingly, our human results demonstrated that the increase in muscle glycogen replenishment throughout the recovery period (Figure 1c) observed with GA supplementation (2000 mg) did not involve the up-regulation of GLUT4 in muscle (Figures 1d and Figure 4c). It is well established that GLUT4 is responsible for glucose utilization in muscle, which among other stimuli, is affected by physical activity. One way in which this has been demonstrated is by the upregulation of GLUT4 gene expression in response to a single bout of cycling exercise (75% VO_2max_ for 60 min) [27]. Furthermore, a study by Biensø et al.. (2012) subjected young healthy male participants to 45 min of single-leg exercise. Muscle samples before and after exercise were analyzed for GLUT4 and glycogen. Their results showed a positive correlation between skeletal muscle glycogen synthesis and the expression of GLUT4 protein in muscle [28]. Surprisingly, our study showed that the rise in muscle glycogen capacity due to GA supplementation did not result from the increase in GLUT4 in muscle. This inconsistency between studies could be due to timing and/or our experimental technique. Thus, it’s possible we missed quantifying GLUT4 redistribution from intracellular stores to the plasma membrane in response to post-exercise garlic supplementation during the 3-hour period. It’s also reasonable to believe that these differences were because we relied on an immunohistochemistry assay rather than immunofluorescence attaining and microscopy [29]. Additionally, in a previous human trial conducted by Anim-Nyame et al.. (2004), healthy female participants were administered garlic supplement tablets (600 mg/day) for 7 days [30]. The study showed a significant correlation between garlic supplementation and blood flow, as well as plasma IL-6 levels (*r* = 0.8, *p* = 0.001). Studies have reported that blood interleukin 6 (IL-6) can aid in the clearance of calcium ions in the sarcoplasmic reticulum of muscles and promote the relaxation of vascular smooth muscles [31,32]. Thus, it is possible that garlic supplementation may increase blood flow in the body, which could be attributed to the enhanced IL-6 response in the blood.

In the current human study, we observed that GA supplementation significantly lowered blood glucose levels compared to placebo at 30- and 120-min post-exercise (Figure 1a, *p* < 0.05). Furthermore, our study showed that muscle glycogen replenishment was significantly higher with GA supplementation compared to placebo at 3-h post-exercise (Figure 1c). Therefore, it is possible that GA supplementation may have increased the flow of blood (blood flux) during the 3-hour recovery period post-exercise [30]. The improved blood flow may have led to accelerated muscle glucose utilization and glycogen synthesis rates, even though GLUT4 translocation did not reach threshold/saturation. Unfortunately, we were unable to measure blood flow directly or heart rate measurement as indirect flow, and determine blood IL-6 concentrations during the recovery period in the present study. Because of this, we cannot provide direct evidence that muscle blood flow was affected by garlic supplementation. On the other hand, our study found that GA supplementation significantly lowered glucose area under curve (GAUC) compared to the placebo group at 30–120 min post-exercise (GAUC 30–120 min, GA:171.7 ± 75.82; Placebo: 321.7 ± 64.13, *p* < 0.05). Therefore, we suggest that GA supplementation has the capacity to significantly increase muscle glucose utilization throughout the recovery period. Thus, our findings suggest that high-dose GA supplementation consumed immediately after exercise can enhance muscle glycogen replenishment by promoting glucose utilization in the muscle, possibly through the acceleration of blood flow. In addition, samples from this study were analyzed using immunohistochemistry, which could miss the processes of GLUT4 translocation to the plasma membrane. This would be a reasonable explanation as to why we didn’t observe alterations in glucose transporter expression during the 3-hour recovery period. Because we observed higher glycogen content, it’s likely that we missed detecting this process due to the wide sampling timeframe.

Tsao et al. recently conducted a single-blind crossover study for 4 weeks to evaluate the effects of high-dose garlic supplementation (1000 mg/day) on reducing oxidative stress of MDA, TNF-α, and LDH. Although the investigation found that supplementation attenuated oxidative stress, this improvement occurred in the absence of changes to whole-body energy metabolism during a 40-km cycling exercise [33]. Related to this, Mohammadnia et al. reported no impact on metabolic rate or substrate oxidation when inactive women took 600 mg of garlic supplements before a HIIE exercise challenge (1-minute at 90% VO2max followed by 2-minute rest intervals at 60% VO2max) [34]. In the present study, we observed a significant increase in the concentrations of NEFA and glycerol in the blood immediately after exercise. However, both the GA and placebo groups showed a significant decrease in the concentrations of NEFA and glycerol during the 180-min recovery period, which may be attributed to the consumption of a high glycemic index carbohydrate meal along with the garlic supplementation post-exercise. Regarding RER, garlic extract appeared to increase the calculated carbohydrate oxidation, which was significant at 120 minutes between the two groups (Figure 2c, *p* < 0.05; Table 3). To expand on this, we obtained muscle samples during the 0–3 hour recovery period and assessed the expression of the fatty acid oxidation enzyme ATP-citrate lyase, as well as citrate synthase, an important enzyme that connects carbohydrate to lipid metabolism (Figure 4(a,b), *p* > 0.05). We found that the area under the carbohydrate oxidation curve during 3-h recovery showed no significant difference between GA and placebo trials (GA:7.05 ± 1.44 g/min x 3 h; PLA:6.14 ± 0.09 g/min x 3 h, *p* > 0.05). Based on the AUC in the carbohydrate oxidation curve and gaseous response at 120-min and IHC results from muscle samples, we assume that the effect is quite small over the three-hour period following garlic supplementation. Taken together, we concluded that our protocol, in which participants consumed high GI carbohydrates with GA supplementation immediately after exercise, may have created the conditions for garlic-induced insulin sensitivity [35]. Given our results for muscle glycogen replenishment following post-exercise GA supplementation, garlic-induced insulin sensitivity may have a more pronounced physiological effect on glycogen synthesis than it does on carbohydrate oxidation. Regardless, more muscle physiological studies are needed to evaluate the post-exercise GA effect on carbohydrate metabolism in humans.

In this study, utilizing a protocol consisting of 60-min cycling exercise at 75% VO_2max_, we demonstrated that strenuous exercise significantly increased PGC-1α gene expression in human muscle, regardless of whether the participants had taken GA or placebo. This effect was stronger than the impact of GA on PGC-1α gene expression (Figure 3a). The result of PGC-1α is consistent with the results of previous human exercise studies by Granata et al., (2016), Little et al., (2010), and Huang et al., (2020) [20,36,37]. However, the post-exercise garlic supplementation was unable to significantly change the expression of genes relating to mitochondrial biosynthesis in muscle cells, including SIRT1, ERRα, NFR1, NFR2, MFN1, MFN2, OPA1, Beclin-1, and DRP1 (as shown in Figure 3b & 3C & 3D). Only TFAM (mitochondrial transcription factor) and FIS1 (mitochondrial fission protein 1) for muscle cell mitochondrial biosynthesis showed a significant improvement after post-exercise garlic supplementation (Figure 3(b,c)), *p* < 0.05). Exercised-induced gene expressions of NFR1, TFAM, and downstream gene expressions in our study are inconsistent with the Islam et al. human study (2020), they reported significant positive effects of both a single bout of Tabata cycling and a 4-week Tabata cycling program on NRF-1, NRF2 mRNA expressions, downstream genes related to mitochondrial biosynthesis, and increased NRF-1, and TFAM protein content after four weeks Tabata training [38]. Therefore, the higher intensity Tabata exercise mode induced mitochondrial biogenesis transcript factors rather than those induced by the exercise protocol under 60-min cycling at 75% VO_2max_ in the present study. Interestingly, the administration of GA supplementation immediately after exercise resulted in a significant increase in the expression of TFAM (a transcription factor) and F1S1 (a cleavage factor) genes related to mitochondrial DNA gene expression in muscle, compared to the placebo group (Figure 3(b,c)), *p* < 0.05). Here, we provide evidence to suggest that post-exercise GA supplementation can have a significant impact on the expression of transcription factor TFAM and cleavage factor FIS1 for mitochondrial DNA gene expression. It’s reasonable that diallyl disulfide, which is an organ-sulfur compound in garlic with antioxidant properties [39], effects the gene expression in mitochondrial biogenesis. This has been reported in several mouse studies investigating the positive effects of garlic’s antioxidant phytochemical agent on mitochondrial function. These studies demonstrated that garlic could activate SIR-3 to protect mitochondrial function in diabetic animals and that the diallyl disulfide from garlic can activate the eNOS-Nrf2-Tfam pathway to enhance mitochondrial biogenesis in the mouse myocardium [40,41]. However, although supplementation with garlic after exercise significantly increased the expression of TFAM and FIS1 for mitochondrial DNA gene expression in human muscle, there was no significant change in the expression of other mitochondrial biogenesis-related genes, including SIRT1, ERRα, NFR1, NFR2, MFN1, MFN2, OPA1, and Beclin-1, DRP1. Therefore, the potential physiological effect of upregulating the expression of mitochondrial biogenesis transcription factors in human skeletal muscle cells after exercise through post-exercise supplementation with 2000 mg of garlic remains to be elucidated and requires further studies for a better understanding.

With any study design, there are some limitations that need to be addressed and considered for future work. For example, analyzing only a small biopsied muscle sample from a large muscle may not fully represent the entirety of that muscle. As well as the semi-quantitative method of PAS stain on glycogen content may not have been ideal. Therefore, future studies should consider alternative techniques for obtaining human muscle samples. Modifications to improve PAS staining for quantitative analyses of muscle glycogen is also called for. Lastly, it may not be feasible to assume that the benefit of GA supplementation observed in this study with active college students can be applied to elite athletes. So we recommend using elite athletes for future studies to better focus on the potential ergogenic properties of post-exercise glycogen replenishment after GA supplementation.

In terms of sports competition, increasing glycogen concentration in skeletal muscle before competition can significantly improve endurance performance and mitigate fatigue-related physiological factors during exercise [42]. Therefore, this study reported here demonstrated that a post-exercise high-dose (2000 mg) oral GA supplement can enhance muscle glycogen replenishment throughout the recovery period. However, immediate post-exercise garlic supplementation intake was unable to upregulate mitochondrial biogenesis-related gene expression in muscle. In conclusion, and to the best of our knowledge, we are the first to show that post-exercise GA supplementation has ergogenic potential, specifically via enhanced replenishment of muscle glycogen. Improved restoration of muscle glycogen will undoubtedly benefit athletic performance involving continuous high-intensity exercise. Thus, additional studies are warranted to further elucidate the underlying mechanisms of post-exercise GA supplementation and its potential ergogenic effects for athletes.

## Data Availability

The results of the current study are presented clearly, honestly, and without fabrication, falsification, or inappropriate data manipulation, and the data that support the findings of this study are available from the corresponding author upon reasonable request.
